# Fluorine impairs carboxylesterase 1-mediated hydrolysis of T-2 toxin and increases its chondrocyte toxicity

**DOI:** 10.3389/fnut.2022.935112

**Published:** 2022-08-03

**Authors:** Yumeng Jia, Sirong Shi, Bolun Cheng, Shiqiang Cheng, Li Liu, Peilin Meng, Xuena Yang, Xiaoge Chu, Yan Wen, Feng Zhang, Xiong Guo

**Affiliations:** Key Laboratory of Trace Elements and Endemic Diseases of National Health and Family Planning Commission, School of Public Health, Health Science Center, Xi'an Jiaotong University, Xi'an, China

**Keywords:** fluorine, carboxylesterase 1, hydrolysis, T-2 toxin, chondrocyte, Kashin-Beck disease

## Abstract

**Background:**

T-2 toxin is recognized as one of the high-risk environmental factors for etiology and pathogenesis of Kashin-Beck disease (KBD). Previous evidence indicates decreased serum fluorine level in KBD patients. However, whether fluoride could regulate carboxylesterase 1 (CES1)-mediated T-2 toxin hydrolysis and alter its chondrocyte toxicity remains largely unknown.

**Methods:**

In this study, *in vitro* hydrolytic kinetics were explored using recombinant human CES1. HPLC-MS/MS was used to quantitative determination of hydrolytic metabolites of T-2 toxin. HepG2 cells were treated with different concentration of sodium fluoride (NaF). qRT-PCR and western blot analysis were used to compare the mRNA and protein expression levels of CES1. C28/I2 cells were treated with T-2 toxin, HT-2 toxin, and neosolaniol (NEO), and then cell viability was determined by MTT assay, cell apoptosis was determined by Annexin V-FITC/PI, Hoechst 33258 staining, and cleaved caspase-3, and cell cycle was monitored by flow cytometry assay, CKD4 and CDK6.

**Results:**

We identified that recombinant human CES1 was involved in T-2 toxin hydrolysis to generate HT-2 toxin, but not NEO, and NaF repressed the formation of HT-2 toxin. Both mRNA and protein expression of CES1 were significantly down-regulated in a dose-dependent manner after NaF treatment in HepG2 cells. Moreover, we evaluated the chondrocyte toxicity of T-2 toxin and its hydrolytic metabolites. Results showed that T-2 toxin induced strongest cell apoptosis, followed by HT-2 toxin and NEO. The decreased the proportion of cells in G0/G1 phase was observed with the descending order of T-2 toxin, HT-2 toxin, and NEO.

**Conclusions:**

This study reveals that CES1 is responsible for the hydrolysis of T-2 toxin, and that fluoride impairs CES1-mediated T-2 toxin detoxification to increase its chondrocyte toxicity. This study provides novel insight into understanding the relationship between fluoride and T-2 toxin in the etiology of KBD.

## Introduction

Over the past century, the explosive development of the fluorochemical industry has led to a substantial increase in the emission of fluorine-containing compounds in the biosphere (1). In addition, the widespread use of fluorinated drugs, fluorinated products, and agrochemicals further endangers human health and environmental safety (1, 2). Fluorine, one of the essential trace elements for human beings, plays an important role in growth and development, bone metabolism, and reproduction. Appropriate amount of fluoride is beneficial to the formation of teeth and the deposition of bone calcium (3, 4). However, excessive intake may lead to fluorosis, and fluorine deficiency may cause a variety of physiological and pathological changes (5, 6). According to an earlier epidemiological study, in areas with low fluoride, more people are suffering from osteoporosis (7). Kashin-Beck disease (KBD) is an endemic chronic osteochondropathy that mainly damages to articular cartilage and epiphyseal plate cartilage. Recent investigations have identified that level of serum fluorine in KBD patients was substantially lower than that of healthy individuals (8, 9). Therefore, both the deficiency and excess of fluoride will lead to osteochondral-related diseases.

T-2 toxin is the most toxic fungal secondary metabolite majorly produced by *Fusarium* trichomoniae (10). It mainly contaminates foods such as wheat, barley, rye, oats, and corn. After eating contaminated cereals and feed, human and animals will lead to gastrointestinal, skin, thyroid, bone marrow, and red blood cell damages. T-2 toxin is considered as one of the high-risk environmental factors for etiology and pathogenesis of KBD. Epidemiological studies and meta-analysis reviewed that the positive detection rate of T-2 toxin and the overall positive detection rate of the T-2 toxin with concentration more than 100 ng/g were slightly higher in KBD areas than that in non-KBD areas (11). Moreover, a low nutrition diet and exposure to T-2 toxin is firstly established as the KBD experimental animal model (12–14). In this Sprague-Dawley rat model, epiphyseal plates exhibited chondrocyte necrosis, which is one of the most typical phenotypes for KBD (12). In addition, numerous *in vitro* studies uncovered that T-2 toxin-induced chondrocyte injury by regulating the expression levels of Wnt/β-catenin pathway (15, 16), SECISBP2-mediated selenoprotein (17), IGFBP2 (18), heat shock protein 47 (19), and integrin α2 (20), involving in the pathogenesis of KBD. The potential mechanisms are involved in mitochondrial injury, apoptosis, oxidative stress, extracellular matrix metabolic disorders, such as the abnormal expression of MMPs/TIMP-1, type II collagen, and core proteoglycan.

**Figure 1:**
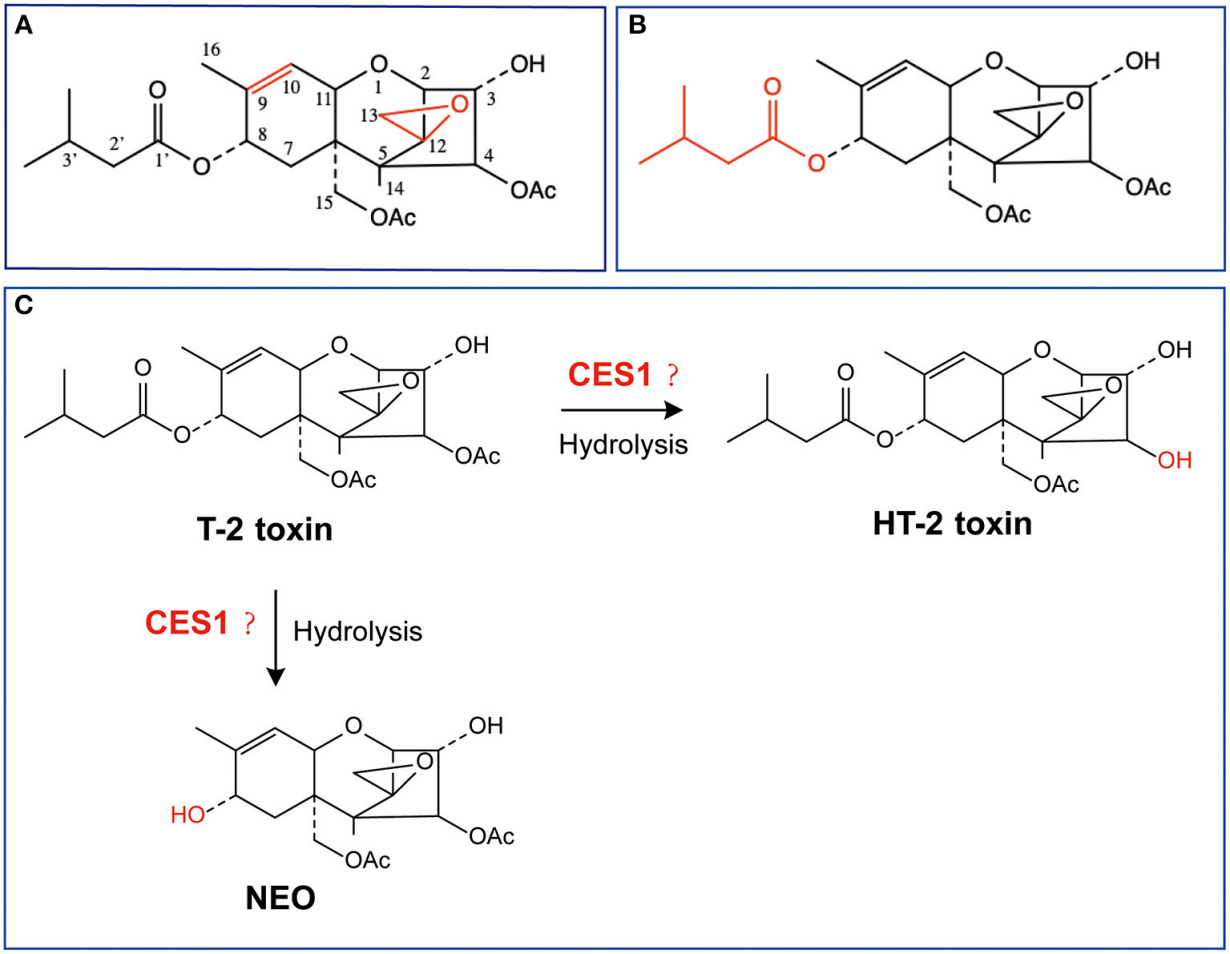
Chemical structures of T-2 toxin and its putative hydrolytic metabolic pathways. **(A)** Toxic group; **(B)** Acetyl group; **(C)** Possible hydrolytic metabolic pathways. NEO, neosolaniol.

Once exposure or oral intake of T-2 toxin, it undergoes a series of metabolic processes including hydrolysis, oxidation, and acetylation in the liver, with the prototype cannot be detected after 30 minutes (10, 21). Until now, more than 20 kinds of T-2 toxin metabolites have been identified, including HT-2 toxin, neosolaniol (NEO), 3'-OH-T-2 toxin, T-2 triol, and acetyl T-2 toxin. Among which, HT-2 toxin is the most important and dominant metabolite that can be detected *in vivo* (22). On the one hand, compelling chemical evidence validates that the toxicity of T-2 toxin is closely related to its chemical structure, in which C12, C13 epoxy ring and C9, C10 double bond are the toxic functional groups of T-2 toxin (refer Figure 1) (23). Ring opening reaction or double-bond reduction reaction of epoxy ring can reduce the toxicity of T-2 toxin. Through structural analysis, we found that most of the T-2 toxin metabolites retained the maternal nuclear structure. Thus, we speculated that the metabolites of T-2 toxin exhibited approximate chondrocyte toxicity to the prototype. On the other hand, considering the fact that CES-mediated hydrolysis usually contributing to detoxification of many environmental toxicants or carcinogens (24), the hydrolysis metabolites of T-2 toxin may display attenuated chondrocyte toxicity. As one of the subtypes of CES, CES1 is highly expressed in the liver and prefers to hydrolyze substrates with smaller ethanol groups and larger acetyl groups (25), which is consistent with the structure of T-2 toxin. Therefore, it is supposed that CES1 is responsible for T-2 toxin hydrolysis. Up to now, limited studies focused on whether fluoride could regulate CES1-mediated hydrolysis of T-2 toxin and alter the chondrocyte toxicity of its metabolites. To further clarify such an ambiguous relationship between fluoride and T-2 toxin metabolism, we directly compared whether sodium fluoride could affect the expression level and activity of CES-mediated hydrolysis. In addition, we evaluated the chondrocyte toxicity of T-2 toxin and its hydrolytic metabolites. Our study provides novel insight into understanding the relationship of suspected etiology of KBD.

## Materials and methods

### Chemicals and reagents

T-2 toxin, HT-2 toxin, and NEO were all purchased from J&K Scientific (Beijing, China). Human CES1 bactosomes that expressed in *Escherichia coli* was purchased from Cypex Ltd. (Scotland, UK). Sodium fluoride (NaF) was purchased from Sigma-Aldrich (St. Louis, MO, USA). High-performance liquid chromatography (HPLC)-grade methanol was the product of Merck (Darmstadt, Germany). Formic acid and other chemicals and solvents used were of analytical grade or above and were purchased from commercial resources. Deionized water was purified using a Milli-Q system (Millipore, Milford, MA, USA).

### Cell culture and treatment

HepG2 cells were purchased from the Shanghai Institute of Cell Biology at the Chinese Academy of Sciences (Shanghai, China). HepG2 cells were cultured in Dulbecco's modified Eagle's medium (DMEM; HyClone, Logan, UT, USA) supplemented with 10% fetal bovine serum (FBS; ExCell Bio Co., Ltd. Shanghai, China), penicillin/streptomycin, and 1× nonessential amino acids. Cells were incubated at 37°C in a humidified atmosphere of 5% CO_2_. To explore whether NaF affects CES1 expression *in vitro*, cells were seeded in 6-well plates, and treated with NaF at a concentration of 0, 1, 10, 100, and 1,000 μM.

C28/I2 cells were seeded and cultured in 6-well plates, and incubated with DMEM supplemented with 10% FBS. To illustrate the difference of chondrocyte toxicity of T-2 toxin and its hydrolytic metabolites, C28/I2 cells were treated with different concentrations of T-2 toxin, HT-2 toxin, and NEO, and then the cell viability, apoptosis, and cell cycle were determined.

### Kinetic analysis of T-2 toxin hydrolytic activity in recombinant CES1

T-2 toxin hydrolase activity in recombinant human CES1 was determined as follows. The typical incubation mixture (at a final volume of 200 μL) contained 100 mM potassium phosphate buffer (pH 7.4) and enzyme source (recombinant human CES1: 0.01mg/mL). The organic reagent in the incubation mixture was <1% in the final concentration. After 5 min of preincubation at 37°C, reaction was initiated by the addition of T-2 toxin (1–100 μM). The reaction was terminated by the addition of 100 μL of ice-cold acetonitrile after a 5 min incubation.

Kinetic parameters were estimated from curves fitted to the data by non-linear regression analysis using the Michaelis–Menten equation, *v* = V_max_ × [S]/(K_m_ + [S]). (Prism, GraphPad Software Inc., San Diego, CA, USA), where *v* is the velocity of the reaction, V_max_ is the maximum velocity, S is the substrate concentration, and K_m_ is the Michaelis constant.

### LC-MS/MS determination of T-2 toxin and its hydrolytic metabolites

Liquid chromatography-tandem mass spectrometry (LC-MS/MS) system was acquired using an LC-20AD liquid chromatographic system (Shimadzu, Kyoto, Japan) coupled to a Triple Quad 3200 mass spectrometer (AB Sciex). Analyst 1.5.1 software was used for data analysis. The calibration curve concentration ranged from 0 to 1,000 nM for HT-2 toxin. Separation was performed on Shim-pack VP-ODS column (2.0 mm i.d. × 150 mm) at 40°C. The flow rate was 0.2 mL/min. The mobile phases consisted of solvent A (5 mM ammonium acetate in 0.1% formic acid in water) and solvent B (0.1% formic acid in methanol). The optimal gradient elution process was set as follows: phase B was increased from 65 to 90% during the period of 0 to 2 min, and maintained for 2 min, and then declined to 65% during the period of 4–5 min, and kept unchanged until the end of elution at 7 min. Typical MS conditions are as shown in Table 1 for the determination of T-2 toxin its hydrolytic metabolites by multiple reaction monitoring in positive ion mode.

**Table 1:** Mass spectrometric settings for quantification of T-2 toxin, HT-2 toxin, and NEO.

**Compound**	**Parent ion (*m/z*)**	**Daughter ion (*m/z*)**	**Declustering potential (V)**	**Collision energy (V)**	**Collision cell exit potential (V)**
T-2 toxin	483.6	215.2	42	26	32
HT-2 toxin	441.7	215.2	28	17	15
NEO	399.7	185.2	30	28	24

*NEO, neosolaniol*.

### RNA isolation and real-time quantitative RT-PCR (qRT-PCR)

To determine whether NaF could modulate the expression level of CES1, we extracted total RNA from HepG2 cells using Trizol reagent (Invitrogen, Carlsbad, California, USA) according to the manufacturer's instructions and were performed as described elsewhere (26, 27). The following primer sequences were used: for *CES1*: 5′- AGTTTCAGTACCGTCCAAGC-3′ (forward) and 5′- CCATCTTGCTAAGTCTGATCTCC-3′ (reverse); for *GAPDH*: 5′- GCACCGTCAAGGCTGAGAAC-3′ (forward), and 5′- TGGTGAAGACGCCAGTGGA-3′ (reverse). The cycle of threshold (Ct) for each sample was normalized to that of *GAPDH*. The results were then analyzed using the comparative ΔΔCt method [2(^−Δ*ΔCt*^)] for relative quantification the expression of target gene, where ΔCt = Ct (target) – Ct (GAPDH).

### Western blotting analysis

Western blotting analysis was used to further confirm the alteration in the mRNA expression level of *CES1* after the NaF treatment in HepG2 cells. Total protein was extracted using RIPA lysis buffer (KeyGEN Bio TECH, Nanjing, Jiangsu, China) supplemented with 1% protease inhibitor cocktail (Thermo, Rockford, IL, USA). The supernatants were transferred and quantified by BCA Assay Kit (Tiangen Biotech, Beijing, China). Lysates (protein of 50 μg each) were separated by 10% SDS-PAGE and transferred to PVDF membrane (Amersham Pharmacia Biotech, Uppsala, Sweden). After being blocked with Tween-20 containing 5% (w/v) non-fat dry milk for 1 h at room temperature, the membrane was incubated with primary antibodies against CES1 (1:25000; Abcam, Cambridge, MA, USA), cleaved caspase-3 (1:500; Abcam, Cambridge, MA, USA), CDK4 (1:5000; Abcam, Cambridge, MA, USA), CDK6 (1:100000; Abcam, Cambridge, MA, USA), and GAPDH (1:5000; Bioworld Technology, Nanjing, Jiangsu, China) at 4°C overnight. Subsequently, the membrane was incubated with horseradish peroxidase (HRP)-labeled secondary antibody (1:10000; ZSGB-BIO Co., Ltd., Beijing, China) for 1 h at room temperature. Finally, the immunoreactivity was detected by enhanced chemiluminescence HRP substrate system reagent (Millipore Corporation, Billerica, MA, USA). The intensity of the target protein bands was captured and analyzed by Image J software (NIH, Bethesda, MD, USA). The target protein levels were normalized to that of GAPDH.

### MTT assay

In order to evaluate the chondrocyte toxicity of T-2 toxin and its hydrolytic metabolites, C28/I2 cells were seeded in 96-well plates. T-2 toxin, HT-2 toxin, and NEO at the concentration of 0–200 ng/mL were added for the incubation of 12, 24, and 48 h. Then, 20 μL of MTT solution (5 mg/mL prepared with PBS, pH = 7.4) and stock solution was added and continued to incubate for 4 h. After that the culture medium was discarded and 100 μL of dimethyl sulfoxide (Sigma, USA) was added to each well to solve the insoluble formazan crystals. The absorbance was measured in multiplate reader (Infinite M200, Tecan, Männedorf, Switzerland) at the 490 nm wavelength.

### Hoechst 33258 staining

To compare the cell apoptosis after treatment with T-2 toxin and its hydrolytic metabolites, C28/I2 cells were stained with Hoechst 33258 according to the manufacturer's instructions (KeyGEN Bio TECH, Nanjing, Jiangsu, China). Based on the results of MTT assay, C28/I2 cells were plated in a 6-well plate and treated with 20 ng/mL of T-2 toxin, HT-2 toxin and NEO for 24 h. Then, cells were incubated with 5 μL of Hoechst 33258 solution per well and fixed at 37°C for 10 min. Finally, we observe cell apoptosis using a fluorescence microscope, and strong fluorescence was recognized as the nuclei of apoptotic cells.

### Annexin V-FITC/PI assay

The apoptosis rate was further evaluated using Annexin V-FITC/PI according to the manufacturer's instructions (KeyGEN Bio TECH, Nanjing, Jiangsu, China). C28/I2 cells were plated in a 6-well plate and treated with 20 ng/mL of T-2 toxin, HT-2 toxin and NEO for 24 h. Following treatment, the cells were collected and washed with PBS. Subsequently, cells were resuspended in 500 μL of binding buffer. After that, 5 μL of Annexin V-FITC and 5 μl of propidium iodide were added to the cell suspension and incubated for 15 min at room temperature in the dark. Cell fluorescence was assessed by flow cytometry (BD FACSCanto, CA, USA) within 1 h. The percentages of early apoptotic (Annexin V + /PI-) or late apoptotic/necrotic (Annexin V + /PI +) rates were obtained.

### Cell cycle analysis

For the cell cycle analysis, C28/I2 cells were seeded in a 6-well plate and treated with 20 ng/mL of T-2 toxin, HT-2 toxin, and NEO for 24 h. Then, cells were fixed with pre-cooled ethanol for 2 h at 4°C and stained with propidium iodine. Cell fluorescence was assessed by flow cytometry (BD FACSCanto, CA, USA) within 1 h at wavelength of 488 nm.

### Statistical analysis

Statistical analyses were performed through SPSS version 16.0 software (SPSS Inc, USA). The data were expressed as mean ± standard deviation (SD). Differences between two groups were analyzed using Student's *t-*test when exhibited a normal distribution. One-way analysis of variance (ANOVA) was used to compare the differences among groups. A *P-*value <0.05 was considered statistically significant.

## Results

### Sodium fluoride inhibits CES1-mediated T-2 toxin hydrolytic metabolism

**Figure 2:**
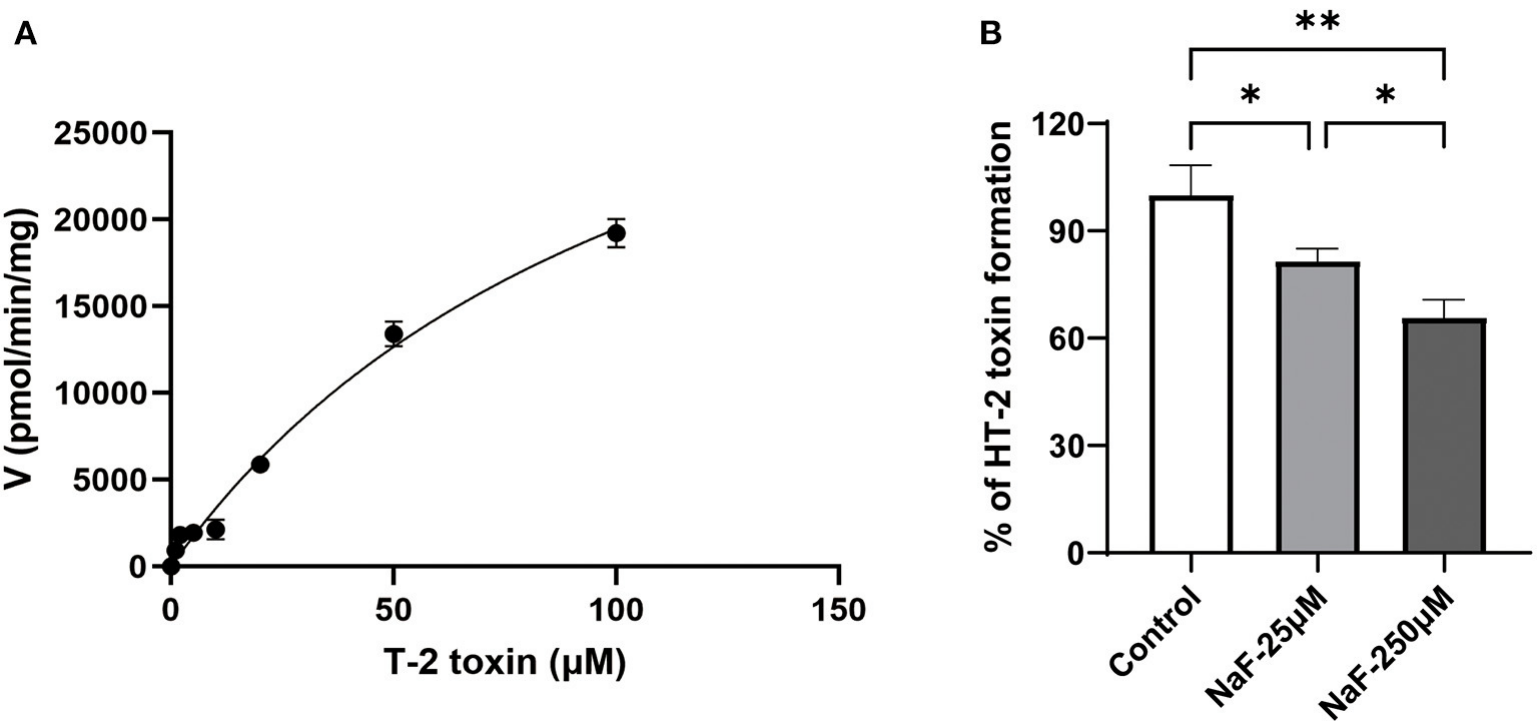
Sodium fluoride inhibits CES1-mediated T-2 toxin hydrolysis. **(A)** Kinetic analysis of T-2 toxin to form HT-2 toxin in recombinant human CES1; **(B)** Sodium fluoride inhibited the generation of HT-2 toxin from T-2 toxin in recombinant human CES1, with the concentration at 25 and 250 μM, respectively. Data are presented as mean ± SD (*n* = 6 each). *P < 0.05, **P < 0.01. NaF, sodium fluoride.

Lin et al. reported that nearly 80% of the hydrolysis of T-2 toxin was significantly suppressed in the presence of CES inhibitors (28), indicating CES was mainly involved in the hydrolytic metabolism of T-2 toxin. In order to identify whether CES1 is responsible for the hydrolysis of T-2 toxin, we used recombinant human CES1 to conduct enzymatic kinetics analysis. The hydrolytic activity T-2 toxin was measured by the generation of HT-2 toxin and calculated using Michaelis–Menten equations (Figure 2). The average K_m_ and V_max_ values of T-2 toxin hydrolysis were 116.1 μM, and 42.1 nmol/min/mg protein, respectively. This is the first study to demonstrate that CES1 is involved in T-2 toxin hydrolysis in human. To further explore whether sodium fluoride could affect the hydrolytic activity of CES1 and then subsequently affect T-2 toxin hydrolysis, T-2 toxin was co-incubated with different concentrations of sodium fluoride. We observed that the generation of HT-2 was moderately inhibited by 18.5% when co-incubated with 25 μM sodium fluoride and that was strongly inhibited by 34.3% at the concentration of 250 μM. Taken together, these data suggest that sodium fluoride significantly mediated T-2 toxin hydrolysis and HT-2 toxin formation *via* inhibition of CES1.

### Sodium fluoride dose-dependently down-regulated CES1 at the mRNA and protein levels in hepatocytes

**Figure 3:**
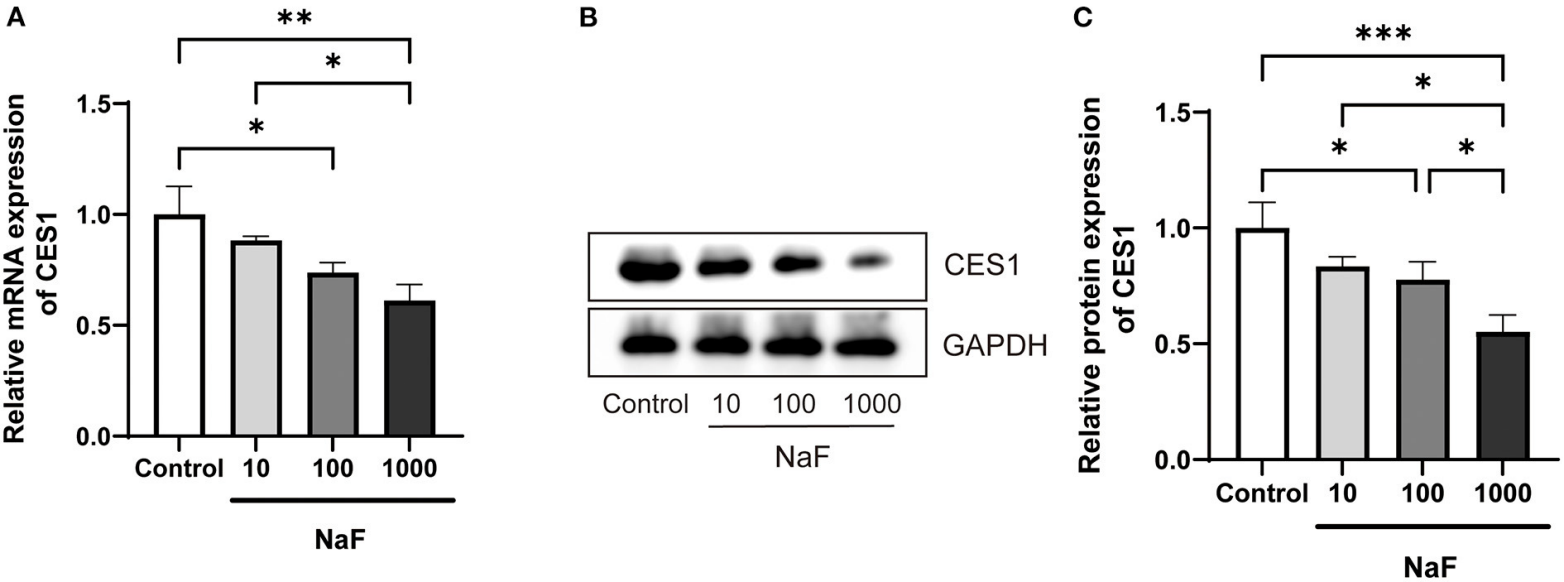
Down-regulated mRNA and protein expression levels of CES1 treated with different concentrations of sodium fluoride in HepG2 cells. **(A)** mRNA expression level of *CES1*; **(B)** The representative immunoblot band of CES1; **(C)** The normalization of CES1 to the internal reference protein GAPDH as control. HepG2 cells were treated with sodium fluoride (10, 100 and 1000 μM) or the same volume of PBS for 24 h. Total RNA was isolated and cell lysates were prepared for qRT-PCR and western blot analysis. Data are presented as mean ± SD (*n* = 6 each). *P < 0.05, **P < 0.01, ***P < 0.001. NaF, sodium fluoride.

Based on the fact that sodium fluoride inhibits the hydrolytic activity of CES1, we further verified whether it could regulate the expression level of CES1. HepG2 cells were treated with sodium fluoride at the concentration of 0, 10, 100, and 1,000 μM. The concentration is according to a previous *in vitro* study (29). The gene and protein expression levels of CES1 were detected by qRT-PCR and western blot analysis, respectively. As shown in Figure 3, after treated with 1,000 μM of sodium fluoride, the gene expression level of CES1 was markedly decreased by 38.9% (*P* < 0.01). 100 μM of treatment moderately down-regulated the gene expression levels of CES1 (*P* < 0.05). Consistent with the observation in mRNA results, protein expression showed a similar trend that sodium fluoride decreased the protein expression levels of CES1 at the concentration of 100 (*P* < 0.05) and 1,000 μM (*P* < 0.001). These data strongly suggest that sodium fluoride down-regulated both the gene and protein expression of CES1 in a dose-dependent manner, which was consistent with the inhibitory effect of sodium fluoride on the hydrolytic activity of CES1.

### Decreased cell viability after T-2 toxin and its hydrolytic metabolites treatment in chondrocytes

**Figure 4:**
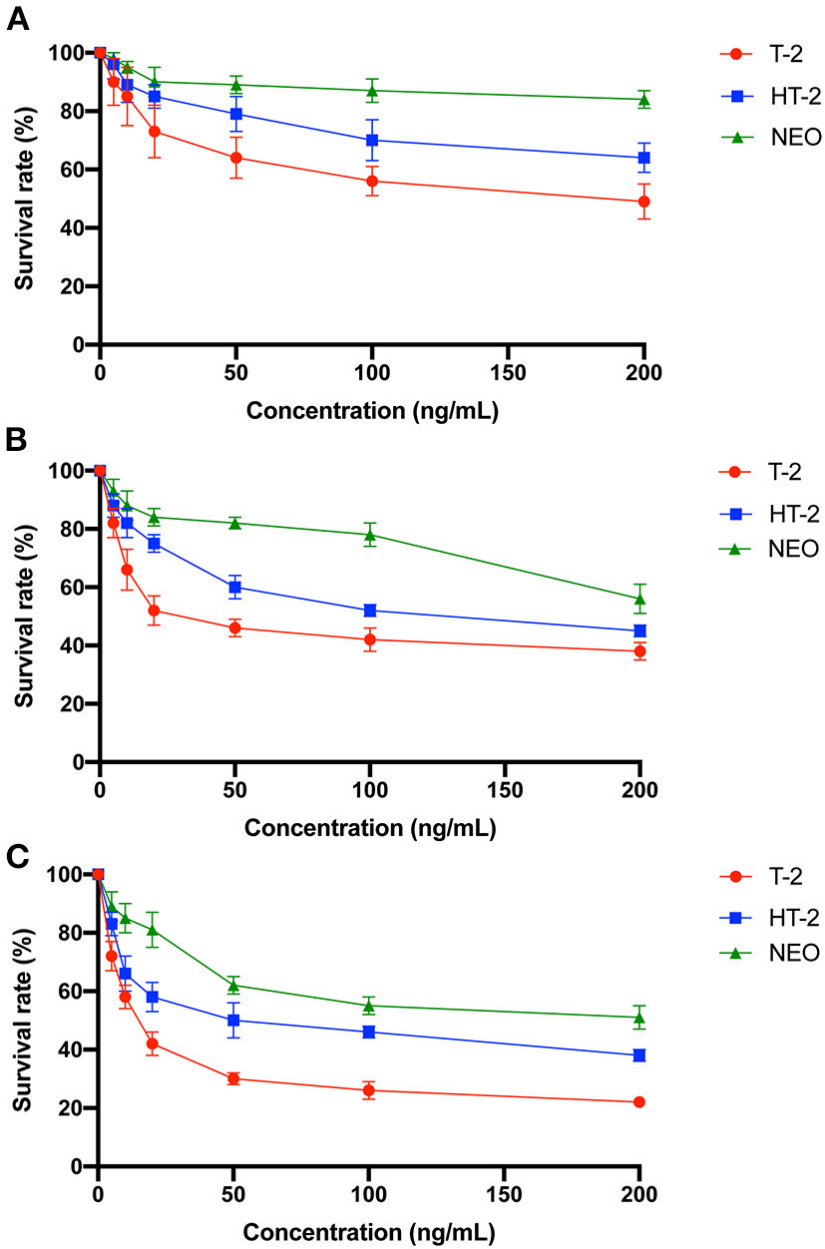
Decreased cell viability of the chondrocytes after treated with T-2 toxin and its hydrolytic metabolites. C28/I2 cells were treated with 0–200 ng/mL of T-2 toxin, HT-2 toxin, and NEO for 12 **(A)**, 24 **(B)**, and 48 **(C)** h. Data are presented as mean ± SD (*n* = 3 each). NEO, neosolaniol.

Since CES1 is generally responsible for the detoxification, we speculate that the chondrocyte toxicity of the hydrolytic metabolites of T-2 toxin is weaker. C28/I2 cells were treated with 0–200 ng/mL of T-2 toxin, HT-2 toxin, and NEO for 12, 24, and 48 h, and MTT assay was used to compare the cell viability. As shown in Figure 4, survival rates showed a significant dose-dependent and time-dependent trend after treatment. Among which T-2 toxin was the most toxic, followed by HT-2 toxin and NEO. 20 ng/mL of T-2 toxin significantly decreased the cell viability by almost half after 24 h. However, the cell viability at the same concentration of HT-2 toxin and NEO was nearly 60% and 80%. These data indicate that the chondrocyte toxicity of T-2 toxin and its hydrolytic metabolites decreased in descending order of T-2 toxin, HT-2 toxin, and NEO.

### Increased cell apoptosis after T-2 toxin and its hydrolytic metabolites treatment in chondrocytes

**Figure 5:**
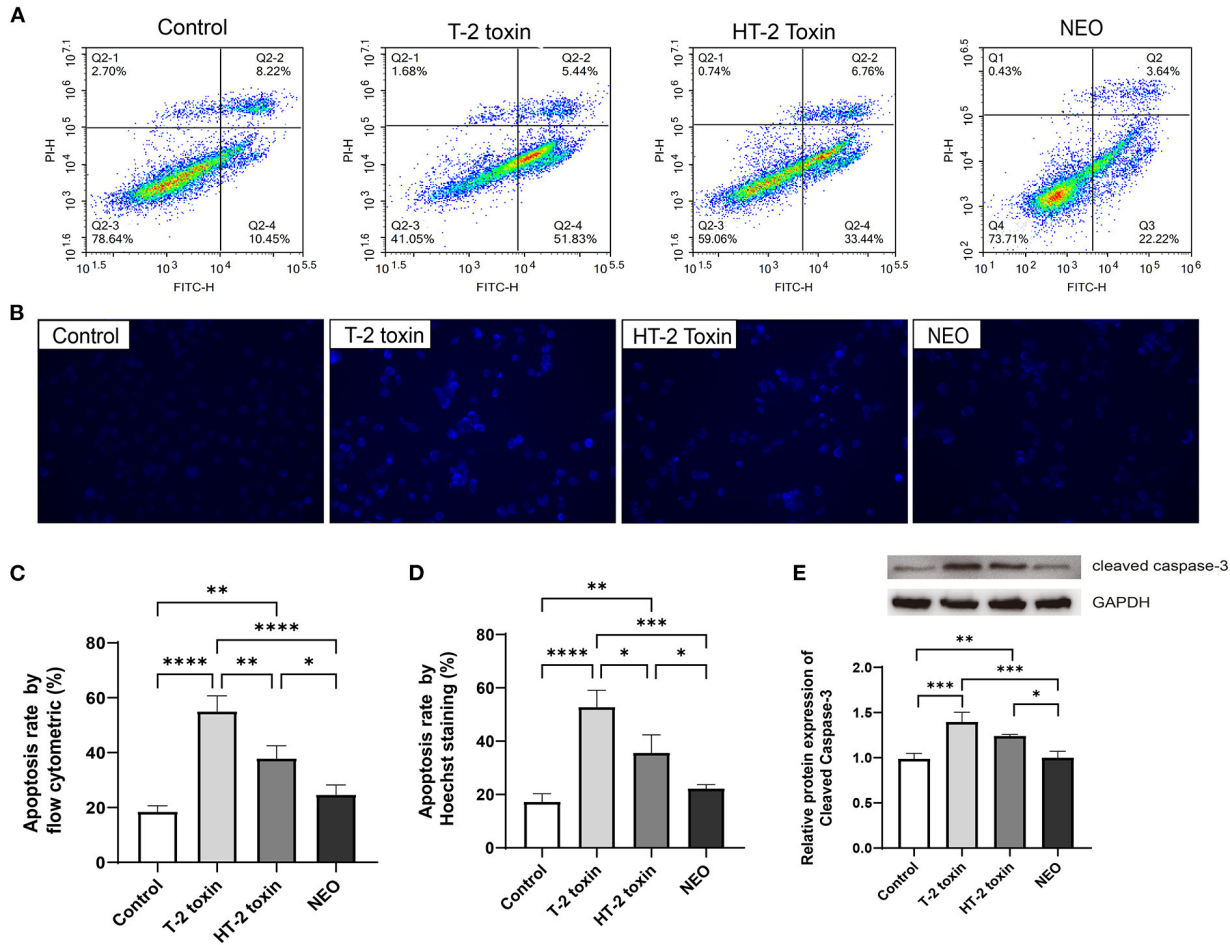
Increased cell apoptosis after treated with T-2 toxin and its hydrolytic metabolites. Apoptosis of chondrocytes was determined by flow cytometry analysis **(A)** and Hoechst 33258 staining **(B)**, the strong fluorescence in the cell nucleus was considered to be apoptosis cells (×400); Apoptosis rates for flow cytometry **(C)** and Hoechst 33258 staining **(D)**; **(E)** Relative protein expression of Cleaved Caspase-3. C28/I2 cells were treated with 20 ng/mL of T-2 toxin, HT-2 toxin and NEO, or the same volume of PBS for 24 h. Data are presented as mean ± SD (*n* = 6 each). *P < 0.05, **P < 0.01, ***P < 0.001, ****P < 0.0001. NEO, neosolaniol.

Cell survival results showed that 20 ng/mL of T-2 toxin, HT-2 toxin, and NEO exhibited appropriate toxic, which was not too much toxic, thus it can be used in the following cell apoptosis experiment. Annexin V-FITC/PI assay by flow cytometry was used to assess the apoptosis rates in chondrocytes. The percentages of early apoptotic (Annexin V + /PI-) or late apoptotic/necrotic (Annexin V + /PI +) rates were obtained and collectively regarded as the apoptosis rates. As shown in Figure 5, compared with the control group, the apoptosis rates of chondrocytes were significantly increased after the treated with of T-2 toxin (*P* < 0.0001) and HT-2 toxin (*P* < 0.01), whereas NEO treatment was comparable. In addition, Hoechst 33258 staining was used for morphological analysis of cell apoptosis, in which strong fluorescence can be observed in the nuclei of apoptotic cells. Consistent with the results of flow cytometry, the strongest fluorescence was observed in T-2 toxin, suggesting the highest cell apoptosis rate (*P* < 0.0001) followed by HT-2 toxin (*P* < 0.01) and NEO. The relative protein expression level of cleaved caspase-3 was significantly up-regulated after treated with T-2 toxin (*P* < 0.001) and HT-2 toxin (*P* < 0.01). These results demonstrate that T-2 toxin leads to markedly cell apoptosis, and its hydrolytic metabolites result in the declined cell apoptosis rate.

### Accelerated cell cycle after T-2 toxin and its hydrolytic metabolites treatment in chondrocytes

**Figure 6:**
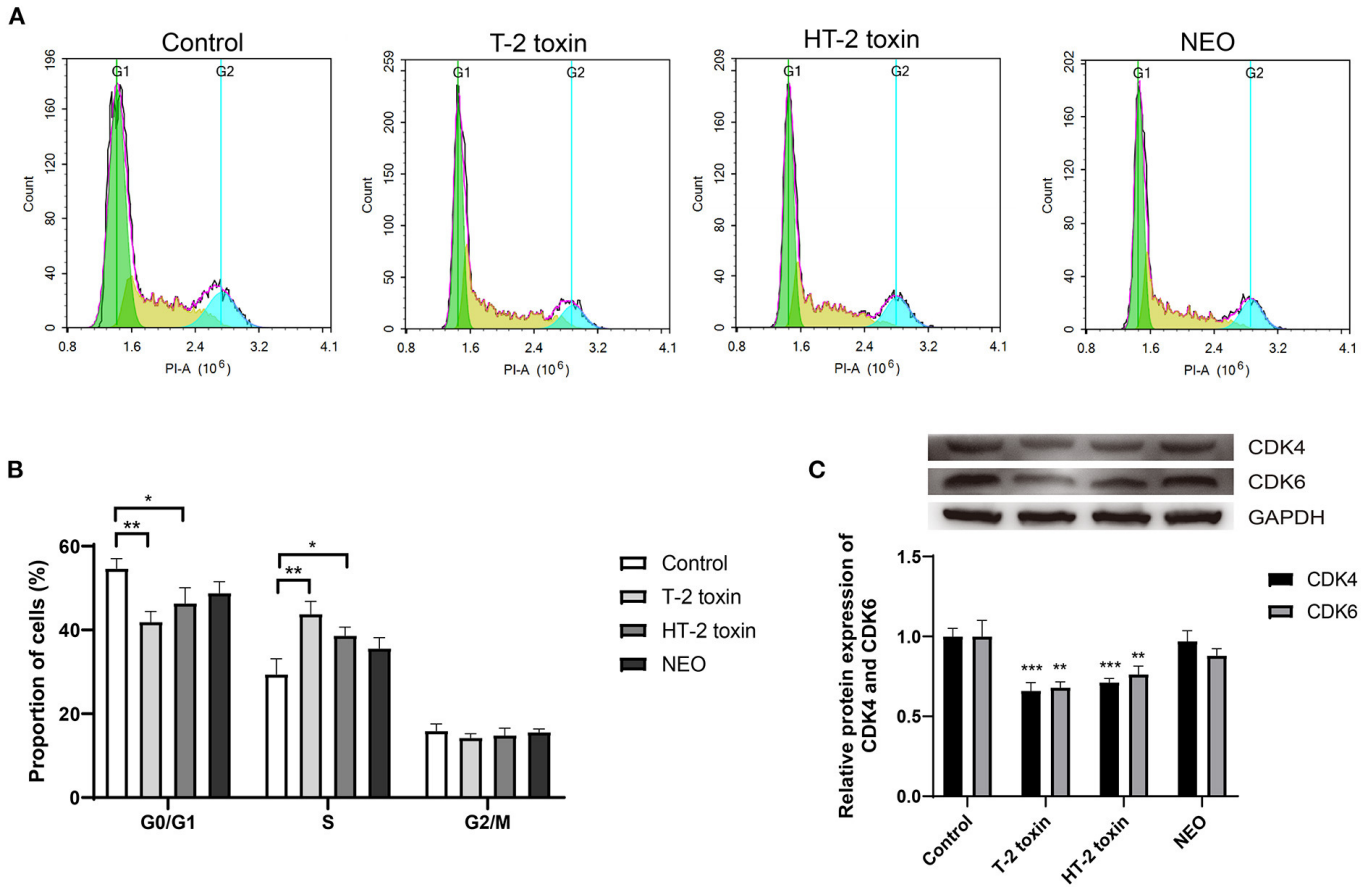
Accelerated cell cycle after T-2 toxin and its hydrolytic metabolites treatment. **(A)** Representative cell cycle results; **(B)** Accelerated cell cycle at the G1 to S phase; **(C)** Relative protein expression of CDK4 and CDK6. C28/I2 cells were treated with 20 ng/mL of T-2 toxin, HT-2 toxin and NEO, or the same volume of PBS for 24 h. Data are presented as mean ± SD (*n* = 6 each). *P < 0.05, **P < 0.01, ***P < 0.001. NEO, neosolaniol.

Although our previous studies indicated that T-2 toxin or HT-2 toxin treatment changed the cell cycle in chondrocytes, the difference between altered cell cycle after treated with T-2 toxin and its hydrolytic metabolites remains unknown. As shown in Figure 6, the proportion of cells in the G0/G1 phase was significantly decreased by 23.4% and 15.2 (both *P* < 0.01) after treated with T-2 toxin and HT-2 toxin, respectively, whereas it was unchanged in NEO group. Meanwhile, the proportion of cells in S phase increased markedly with the highest enhanced proportion after T-2 toxin treatment (*P* < 0.01) and moderately increased after HT-2 toxin treatment (*P* < 0.05). For G2/M phase, there was no marked difference among all the groups. The relative protein expression levels of CDK4 and CDK6 were significantly down-regulated after treated with T-2 toxin and HT-2 toxin. The above results indicate that T-2 toxin and its hydrolyzed metabolites accelerate the cell cycle by reducing the proportion of cells in G0/G1 phase and increasing it in S phase, among which T-2 toxin exhibits the most significant changes.

## Discussion

In this study, we identified that CES1 was involved in the hydrolytic metabolism of T-2 toxin by using recombinant enzyme assay, and then we conducted *in vitro* studies to reveal the regulatory effect of fluoride on the expression of CES1. Moreover, the chondrocyte toxicities of T-2 toxin and its hydrolytic metabolite were evaluated and confirmed in a descending order of T-2 toxin, HT-2 toxin, and NEO. These novel findings would help re-shaping the puzzle of pieces that explain the relationship between fluoride and T-2 toxin for the further investigation of etiology of KBD.

Numerous investigations have indicated that T-2 toxin is a high-risk cause for KBD. Previous evidence revealed that the serum fluorine level in KBD patients was lower than that of healthy control, suggesting fluorine was related to the etiology of KBD (8). However, whether there is a synergistic or antagonistic effect between fluoride and T-2 toxin in the pathogenesis of KBD remains largely unknown. Clinical and experimental evidence suggests that the effect of fluoride on human health mainly depends on its content in drinking water, with low levels less than 0.7 ppm (0.5–1.0 ppm) in drinking water is beneficial and higher levels of more than 1.5 ppm cause deleterious effect on non-skeletal system (including liver). Till now, limited evidence suggests the chronic levels of fluoride that alter the hepatic drug-metabolizing enzymes, including CES1, needs further investigations. In order to illustrate the potential mechanisms, CES1 was used to cross the gap between fluoride and T-2 toxin. We conducted enzymatic kinetic reaction using recombinant human CES1 for quantitative determination the elimination of T-2 toxin and the generation of hydrolytic metabolites, including HT-2 toxin and NEO using LC-MS/MS assay. Our results showed that HT-2 toxin was generated by T-2 toxin when incubated with recombinant CES1 enzyme (Figure 2). However, NEO could not be produced from T-2 toxin in human. Consistent with our results, a previous study conducted by Lin et al. demonstrated the role of the two important phase I drug metabolic enzymes, CYP450 enzymes and CES, in the metabolism of T-2 toxin (28). They observed that only 20% of T-2 toxin was metabolized in the presence of CES inhibitors, while in the presence of both CYP450 enzyme and CES inhibitors, almost all the T-2 toxins was remaining, suggesting a primarily role of CES for the hydrolysis of T-2 toxin to generate HT-2. Our results confirmed and further advanced their results; we identified the subtype CES1 is involved in the hydrolysis of T-2 toxin, while T-2 toxin contains a large acetyl group. Apart from CES1, there are various types of hydrolases, such as CES2, AADAC, PON1, and RKIP in the liver or intestine of human for hydrolytic metabolism (30–34). Certain drug or compound is usually hydrolyzed simultaneously by multiple hydrolases. Hence, our follow-up studies should focus on the discovery of other types of hydrolytic enzymes and their respective contributions for the hydrolysis of T-2 toxin or HT-2 toxin. Except for hydrolysis, *in vivo* studies have indicated that hydroxylation and de-epoxidation are the main metabolic pathways for T-2 toxin (35, 36). In addition, we found that sodium fluoride significantly inhibited the CES1-mediated hydrolytic metabolism of T-2 toxin *via* reducing generation of HT-2 toxin, which is consistent with other hydrolysis reactions that fluoride inhibited the hydrolytic metabolism (37, 38). Taken together, we not only demonstrated that CES1 was responsible for the hydrolysis of T-2 toxin in human for the first time, but also found that sodium fluoride reduced the hydrolytic activity of CES1, thereby attenuating the hydrolysis of T-2 toxin and formation of HT-2 toxin.

In addition to investigating the effect of fluoride on the hydrolytic activity of CES1, we also compared its influence on CES1 expression. Because CES1 mainly exists in the liver, HepG2 cells were treated with different concentrations of sodium fluoride. Results showed that sodium fluoride down-regulated both the gene and protein expressions of CES1 in a dose-dependent manner (Figure 3). We speculate that the possible reason for the decreased expression of CES1 is related to the liver damage caused by fluoride. Fluoride exposure from industrial sources is usually an environmental toxicant. Chronically exposed to excessive fluoride leads to accumulation and toxicity in tissues, including liver, kidney, and brain. Previous study showed that fluorosis could accelerate hepatic oxidative stress in animals, and the mechanism may be related to fluoride-induced hepatocyte apoptosis and autophagy (39–41). In addition, a correlation between the stage of patients with alcoholic cirrhosis and serum fluoride levels was confirmed, with elevated plasma fluoride accompanied by raised alanine aminotransferase and total bilirubin (42). In a recent population-based cross-sectional study, by monitoring fluoride concentrations in plasma and household tap water, as well as evaluating the hepatic parameters in serum, it was found that every 1 mg/mL increase in water fluoride strongly correlated with 0.93 mg/dL decreased blood urea nitrogen concentration (43). Besides, CES1 is a vital drug metabolic enzyme in the liver that encodes about 1% of the total liver genomes (44). The liver inflammation or damage usually leads to alteration of liver drug-metabolizing enzymes, including CES1 (45, 46). Consequently, the above results indicate that fluoride exposure affects the gene and protein expression levels of CES1 *in vitro*.

Due to the fact that CES1 generally mediated the detoxification, T-2 toxin accumulation and T-2 toxin hydrolytic metabolites reduction is deduced to the increased chondrocyte toxicity. Thereafter, we compared the differences of cell viability, apoptosis, and cell cycle of C28/I2 cells after treated with T-2 toxin, HT-2 toxin, and NEO. Consistent with our expectations, we observed significant decreased cell survival rate (Figure 4), increased apoptosis rate (Figure 5), and accelerated cell cycle (Figure 6) after toxin treatment, with the most obvious changes in T-2 toxin, followed by HT-2 toxin and NEO groups. These data confirmed that CES1-mediated hydrolysis of T-2 toxin is a detoxification metabolism. Previous studies in our research group have found that the chondrocyte apoptosis rates induced by T-2 toxin and HT-2 toxin is 49.7 and 36.3%, respectively, which is consistent with our findings (47). The expression levels of apoptosis-related proteins, including Bax, Caspase-9, and Caspase-3 were up-regulated, showing a higher trend in T-2 toxin compared with HT-2 toxin group (47). Cell cycle regulation plays an important role in the proliferation and differentiation of chondrocytes (48, 49). In KBD chondrocytes, there exists abnormal cell cycle (50, 51). Previous evidence has demonstrated that T-2 toxin could induce cell cycle arrest in human primary chondrocytes, and chondrocyte lines, accompanied by alterations in the gene expression of p53 and p21 (50, 52–54). As the main hydrolytic metabolite, a recent study has proved that HT-2 toxin accelerated cell cycle in the differentiating KBD and control human induced pluripotent stem cells, and that it down-regulated both the cell cycle regulation-related genes p21 and cell cycle kinase CDK6 (51). Apart from HT-2 toxin and NEO, both T-2-triol and T-2-tetraol are the hydrolytic metabolites of T-2 toxin that are metabolized by hydrolases. Considering the fact that hydrolytic metabolism is a process to eliminate the toxicity, we speculate that all of the hydrolytic metabolites of T-2 toxin exhibit decreased chondrocyte toxicity.

However, there are still some limitations in our study. First, in order to clarify the whole metabolic pathway of hydrolysis and detoxification of T-2 toxin, other hydrolases except for CES1 that involve in the metabolism of T-2 toxin should be identified. Next, although we carried out *in vitro* study to demonstrate fluoride impairs the expression and activity of CES1, whether CES1-mediated hydrolysis of T-2 toxin was altered under low and high fluoride levels *in vivo* remains elusive, which is a scientific problem worthy of further exploration and clarification. Finally, our results are mainly based on *in vitro* experiments, and thus, conclusion draw from this work has to be interpreted with caution when applying to human study.

## Conclusions

**Figure 7:**
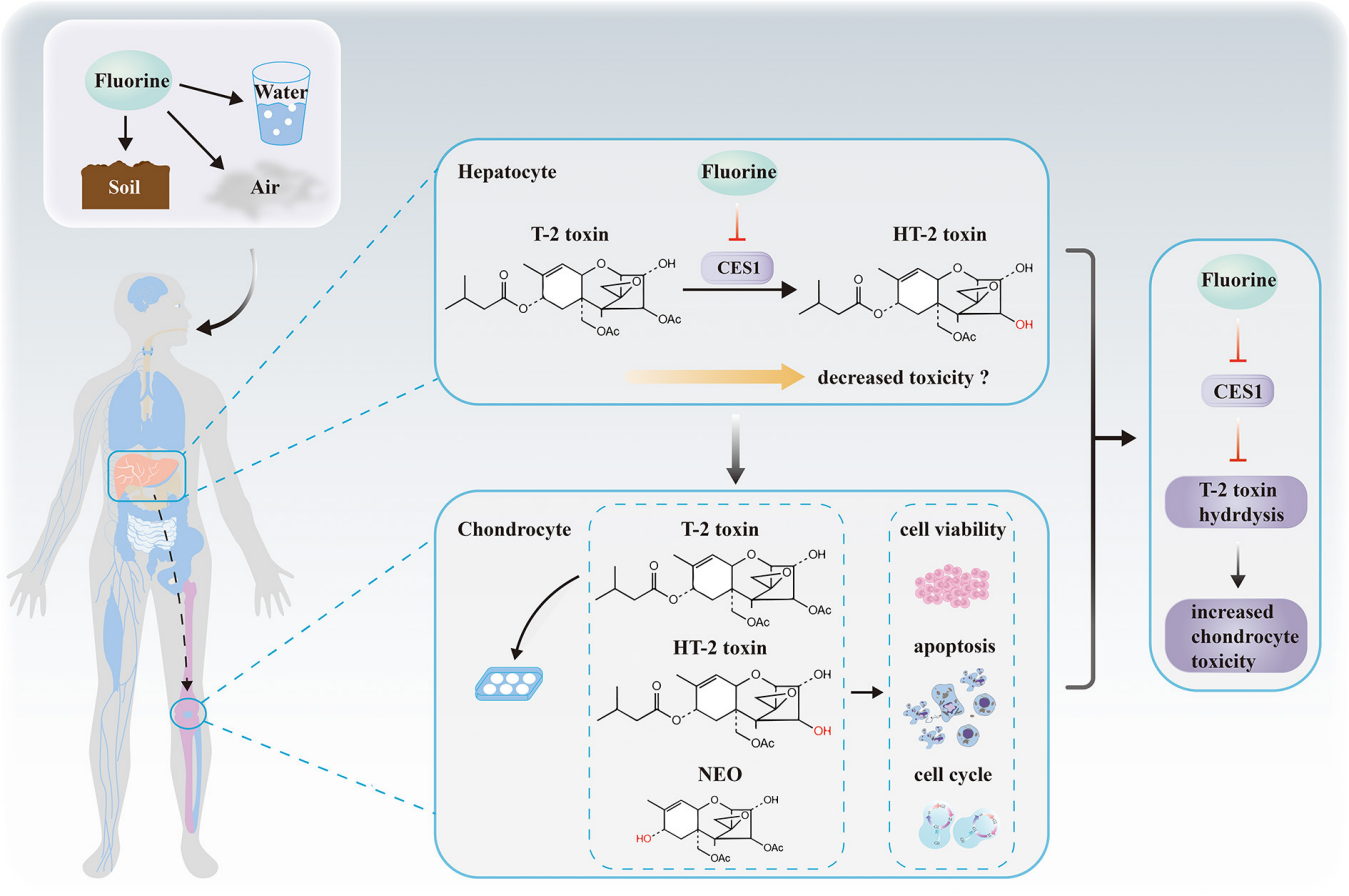
Proposed hypothetical mechanisms revealing the relationship among fluorine, hepatic CES1, T-2 toxin hydrolysis, and chondrocyte toxicity. No causality has been established between the hepatic drug-metabolizing enzymes and chondrocyte toxicity. We suppose that fluorine in the external environment affects the expression and activity levels of CES1 in hepatocytes. Then, we demonstrate CES1 is responsible for the hydrolysis and detoxification of T-2 toxin. Finally, fluoride indirectly decreases the hydrolytic metabolism of T-2 toxin via CES1 and subsequently increases T-2 toxin-induced chondrocyte toxicity.

In summary, to our knowledge, this study is the first to identify that CES1 is responsible for the hydrolysis of T-2 toxin in human, and that fluoride down-regulates the expression and activity of CES1, which ultimately increases the chondrocyte toxicity (Figure 7). Our results decipher the mechanisms underlying the relationship between fluoride and T-2 toxin, which provide a powerful basis for elucidating the etiology of KBD.

## Data availability statement

The original contributions presented in the study are included in the article/supplementary material, further inquiries can be directed to the corresponding authors.

## Author contributions

YJ and FZ designed the study and wrote the manuscript. SS, BC, SC, LL, and PM carried out the experiments and data acquisition. XY, XC, and YW conducted data analysis and interpretation. FZ and XG revised the manuscript. All authors reviewed and approved the final manuscript.

## Funding

This study was supported by the National Natural Scientific Foundation of China (81922059 and 82103959), the Key Projects of international cooperation among governments in scientific and technological innovation (2016YFE0119100), the Natural Science Basic Research Plan in Shaanxi Province of China (2017JZ024), and the Fundamental Research Funds for the Central Universities.

## Conflict of interest

The authors declare that the research was conducted in the absence of any commercial or financial relationships that could be construed as a potential conflict of interest.

## Publisher's note

All claims expressed in this article are solely those of the authors and do not necessarily represent those of their affiliated organizations, or those of the publisher, the editors and the reviewers. Any product that may be evaluated in this article, or claim that may be made by its manufacturer, is not guaranteed or endorsed by the publisher.
